# Mass Spectrometry-Based Analysis of Lipid Involvement in Alzheimer’s Disease Pathology—A Review

**DOI:** 10.3390/metabo12060510

**Published:** 2022-06-02

**Authors:** Andrea R. Kelley

**Affiliations:** Department of Chemistry, United States Air Force Academy, Colorado Spring, CO 80840, USA; andrea.kelley@afacademy.af.edu

**Keywords:** Alzheimer’s disease, amyloid protein precursor, inflammation, oxidative stress, lipid dyshomeostasis, mass spectrometry, mass spectrometry imaging

## Abstract

Irregularities in lipid metabolism have been linked to numerous neurodegenerative diseases. The roles of abnormal brain, plasma, and cerebrospinal fluid (CSF) lipid levels in Alzheimer’s disease (AD) onset and progression specifically have been described to a great extent in the literature. Apparent hallmarks of AD include, but are not limited to, genetic predisposition involving the APOE Ɛ4 allele, oxidative stress, and inflammation. A common culprit tied to many of these hallmarks is disruption in brain lipid homeostasis. Therefore, it is important to understand the roles of lipids, under normal and abnormal conditions, in each process. Lipid influences in processes such as inflammation and blood–brain barrier (BBB) disturbance have been primarily studied via biochemical-based methods. There is a need, however, for studies focused on uncovering the relationship between lipid irregularities and AD by molecular-based quantitative analysis in transgenic animal models and human samples alike. In this review, mass spectrometry as it has been used as an analytical tool to address the convoluted relationships mentioned above is discussed. Additionally, molecular-based mass spectrometry strategies that should be used going forward to further relate structure and function relationships of lipid irregularities and hallmark AD pathology are outlined.

## 1. Introduction

Lipids account for over half of a normal brain’s total dry weight [1]. They play imperative roles in neuronal function, structural development, and energy reserves [2,3]. Some of the primary hallmarks of neurodegenerative disease involve diminishing brain mass [4], cognitive impairment, neuronal death and decay [5], and protein synthesis dysregulation [6], so it comes as no surprise that lipid dysregulation has been linked to several neurodegenerative disease pathologies [2,7,8]. While there exist many biochemical-based methods for the qualitative characterization of lipids in biological systems [9,10,11,12], molecular-based quantitative means of investigating lipid relationships and irregularities in systems continue to be at the forefront of bioanalytical method development. The evolution of lipidomics (a mass spectrometric approach to lipid identification and quantitation in complex samples) [13] and mass spectrometry imaging (MSI) have started to pave the way for advanced means of better understanding some of the convoluted ways that lipid-involved processes might lead to the development and progression of neurodegenerative diseases. Although these methods have led to much progress in the field, there are still advancements to be made. This review outlines the ways in which mass spectrometry-based methods have been used to paint a more complete picture of the most common neurodegenerative disorder, Alzheimer’s disease (AD), as it pertains to some of the most researched mechanisms of lipid involvement in the disease pathology: genetic predisposition involving the apolipoprotein E (APOE) Ɛ4 allele and APOE lipid processing, lipid peroxidation and associated oxidative stress, and inflammation. First, however, it is important to outline the roles of some of the most discussed lipid biomarkers for AD in normal brain homeostasis and how those roles are thought to be altered in the disease state. Additionally, the basics of lipidomics and other lipid-focused mass spectrometry techniques are introduced.

### 1.1. The Role of Lipids in Normal Brain Homeostasis

There are three primary types of lipids found in the brain, each of which is present in close to equal ratios: glycerophospholipids, sphingolipids, and cholesterols [14]. Each of these classes has a specific role in normal brain function, but as a whole, they are responsible for cellular function and development [14]. These lipid classes are discussed later at length as they pertain to mass spectrometry-based implications in disease pathology, but it is first important to understand the basics of their specific roles in normal brain homeostasis.

Phospholipids are incredibly common in the brain and usually contain at least one polyunsaturated fatty acid (PUFA) or monounsaturated fatty acid (MUFA) and one saturated fatty acid [15]. Because of the many different possible combinations of head groups and acyl chains, phospholipids are challenging to analyze [15,16]. In healthy brain homeostasis, phospholipids essentially act as a barrier as part of cell membranes, protecting cells from damage [17]. It has been well documented that fatty acid metabolism is altered with age [18], linking phospholipids to diseases and disorders that are more prevalent in aging populations.

Sphingolipids are prominent membrane lipids and have a multitude of biological functions, including development, toxin reception [19], the regulation of signal pathways, cell interaction and recognition, etc. [14]. They are also very important in myelin (the protective layer around nerves) stability, which is a primary reason why their low expression has been linked to neurodegenerative disease and neuronal death and decay [20].

The brain contains a quarter of all cholesterol in the body [21]. While sphingolipids regulate myelin stability, cholesterol is the key myelin constituent [14]. Important cholesterol synthesis occurs in the central nervous system (CNS) shortly after birth, and any dysregulation in this synthesis can cause significant developmental issues [22]. Lipoproteins transport cholesterol in the CNS [23]. One particular lipoprotein typically linked to cholesterol transport is APOE [24]. Genotypes of this lipoprotein are directly linked to familial Alzheimer’s disease, and thus, issues with cholesterol regulation, synthesis, and transport have also been linked to AD pathology.

While phospholipids, sphingolipids, and cholesterols are not the only lipids implicated in AD pathology, lipids belonging to these three classes are referred to repeatedly in this review and have been the focus of many mass spectrometry-based studies.

### 1.2. Lipids and Mass Spectrometry

Lipidomics is a specialized field of analytical chemistry and bioinformatics dedicated to elucidating the structures, functions, and dynamics of lipids in bodily systems, typically by mass spectrometric methods [25]. Although lipidomics was first described nearly 20 years ago [26], it was not until more recently that the techniques started gaining attention due to the implications of heavy lipid involvement in various neurodegenerative and CNS disease pathways [8,27]. (In the context of this review, any time “lipidomics” is mentioned, it should be assumed that mass spectrometry was utilized as the technique for analysis.) Analytical lipid analysis can be targeted (where the lipids of interest are known) or non-targeted (where all lipids within a sample/system are analyzed without bias). In either case, lipids must first be extracted from the complex sample matrix and prepared for analysis. This can vary from one or two steps to many complex steps involving special method development. (So as to not deviate too far from the scope of the review, the many extraction methods for mass spectrometric lipid analysis are discussed, except where methods were specific to AD research.)

The benefits of utilizing mass spectrometry for lipid detection and quantitation are outlined throughout the review; however, in general terms, mass spectrometry allows for a high-throughput, robust means of analyzing complex samples for lipid content. In addition, absolute quantitation and relative quantitation (the amount of one lipid species compared to another) can be important factors in disease state pathology. Chromatography (a means of separation) can be completed simultaneously with mass spectrometry analysis [28]. While all of these aspects are incredible benefits to neuronal lipid analysis, they all continue to be challenges as more and more information is sought from complex samples. The three main techniques for lipidomics include LC (liquid chromatography)-based, shotgun, and MSI [13]. While the specifics and features of each technique are not within the scope of this review (interested readers can find more information about each in the comprehensive review by Yang and Han [13]), it is important to understand the advantages of and limitations to each technique as we describe them in the context of lipid involvement in AD. LC-based methods, which encompass the majority of the referenced studies in this review, involve chromatographic separation prior to mass spectrometric analysis. LC-based separation has the advantage of being highly selective and able to separate isomers and isobars that are common in lipid classes. However, mobile phase effects and drifting retention times can make the already complex data sets incredibly hard to analyze. Additionally, untargeted lipid analysis is more difficult due to the vast majority of lipid species in complex samples and the lipid-class-specific extraction protocols required for some methods [13].

The term “shotgun lipidomics” was first coined by Han and Gross [29,30]. Both went on to utilize the technique to investigate plasma- and CSF-based lipid involvement in AD. While lipidomics is the study of cellular lipidomes from biological sources, shotgun lipidomics takes it a step further and allows for both the high-throughput identification and quantitation of lipids and also the interaction between varying lipid species and biological systems [31]. While internal standards are required for quantitation, shotgun lipidomic methods are high-throughput, straightforward, and sensitive and allow for large-scale analysis of very complex samples. However, internal standards are often difficult to come by. In the case of using MALDI imaging for shotgun lipidomics, matrix and background interferences in the low mass range can make lipid identification difficult [13].

MSI experiments of specific lipid classes are incredibly informative. Techniques for sample preparation and imaging of lipids are well established [32,33,34,35,36,37]. Tissues can be mapped for localization as well as relative abundance, and imaging data can be combined with data from other modalities to give a clear picture of lipid localization within a particular tissue section. Imaging experiments are typically limited to prominent lipid classes, and fragmentation can occur, making it difficult to identify unknown lipid markers. In addition, data sets for imaging experiments may very well reach over a terabyte, making the analysis portion of the experiment very time-consuming. However, there are programs and statistical analysis software available to be able to more easily accomplish multivariate analyses such as principal component analysis and image fusion [38]. More about each of these topics in relation to specific studies is discussed later.

## 2. Genetic Predisposition Involving the APOE Ɛ4 Allele and APOE Lipid Processing

APOE is a lipoprotein that is integral to lipid metabolism [39]. It is also central in the talk of genetic predisposition to AD and other neurodegenerative disorders. The three major isoforms of APOE are Ɛ2, Ɛ3, and Ɛ4, and each only differs by, at most, two amino acids [40]. It is well researched that having only a single Ɛ4 allele can greatly increase a person’s risk of eventually developing AD [41]. APOE is responsible for the distribution and movement of lipids throughout the body by mediating binding between lipids and cell receptors in plasma [39]. It is not a coincidence that the primary genetic risk factor for developing the disease is involved in lipid metabolism; however, much is still unknown regarding the linkage of the apolipoprotein to neurobiology and neurodegeneration. There are several different types of apolipoproteins, and because mass spectrometry can differentiate between very similar proteins and peptides, it becomes an invaluable tool when investigating genetic disease factors where one species is thought to participate in the onset/progression of disease and another similar species or alternate genotype is thought to protect against the disease. Many studies have identified specific lipid biomarkers for AD (listed earlier); however, few have looked at the ways in which different apolipoproteins and APOE genotypes affect the overall plasma lipidome.

In 2001, DeMattos et al. purified APOE and APOJ (another common apolipoprotein produced by the CNS) from astrocytes extracted from the human APOE transgenic mouse model to determine what made APOE the specific apolipoprotein linked to familial Alzheimer’s disease [42]. It was determined that while APOJ was protein-rich, with phospholipids making up a larger amount of the lipid content than cholesterol, APOE was extremely lipid-rich with nearly equal amounts of cholesterol and phospholipid. This supported the role of APOE, specifically in lipid transport and metabolism [43]. APOJ containing a higher concentration of phospholipids may indicate a more protected cell membrane. APOE being more cholesterol-rich supports the idea that cholesterol metabolism may play a significant role in predisposition to familial Alzheimer’s [44,45]. Utilizing electrospray mass spectrometry, phospholipid content was further classified, with ethanolamine glycerophospholipids being the primary lipid species in both APOE and APOJ. Apolipoproteins purified from CSF proved similar to those purified from astrocytes [42]. This publication proved the usefulness of this purification method to further characterize apolipoproteins via mass spectrometry; however, very few other reports since the early 2000s [46,47] have utilized a method of APOE purification for further experimentation and characterization. It is unclear what other constituents may be colocalized with or near the apolipoproteins. A comparison between the APOE and APOJ environments using imaging combined with mass spectrometry may be able to elucidate further differences between the two that may offer advanced research foci.

Comparison of APOE to other apolipoproteins can yield important information, but maybe even more important to AD pathogenesis research is developing methods to better understand the relationship between APOE genotypes and what makes one seemingly neuroprotective and one predispose someone to neurodegeneration. Just as APOE Ɛ4 is known to be a genetic predecessor for familial Alzheimer’s disease, Ɛ2 has been reported as being neuroprotective [48]. It is suspected that the neuroprotective nature of the Ɛ2 allele may be due to mediation by plasma lipids [49]. A survey of lipidomic studies across multiple cohorts showed a strong correlation between lipid species and the APOE genotype [49]. Furthermore, this correlation seems to be strongest at a younger age, pointing toward potential diagnostic markers for preclinical dementia. Ether lipids have been shown via lipidomic studies to be depleted in advanced disease stages and are thought to contribute to neuroprotection and resilience to neurodegeneration [50]. The lipidomic profiles compared were compiled from mass spectrometry analyses of plasma and serum samples after implementing lipid extraction protocols. In an attempt to clarify the link between the genetic risk factor APOE and more specific lipid biomarkers, Wong et al. quantified lipids in plasma using an untargeted LC-MS method in 152 patients who had no clinical signs of dementia but who did carry at least one Ɛ2 or Ɛ4 allele. It was determined that those who carried the Ɛ2 allele had much higher levels of the phosphatidylethanolamine (PE) phospholipid subclass as well as other phospholipids [51]. Higher levels of some phospholipids have been linked to lower dementia risk [52]. This study was able to demonstrate the link between the two phenomena. A beneficial expansion of this research would focus on the dynamic link of the large phospholipid content to other structures within the brain. This type of research is difficult to accomplish with a single mass spectrometry technique, but multi-modal lipidomics may help to further elucidate the relationships between phospholipids and brain neurochemical pathways in those containing the Ɛ4 allele.

Another phenomenon linking APOE lipid processing to AD involves the importance of the C-terminus of the apolipoprotein in modulating lipid-binding activity. This importance has been characterized [53] in normal brain function. Few have connected modifications present in the AD brain at or near the C-terminus to a dysregulation in APOE lipid binding and transport [54,55]. A 2005 study by Raftery et al. used mass spectrometry to map the major APOE protein kinase CK2 phosphorylation site to Ser296. This correlates to the C-terminus of the apolipoprotein [54]. CK2 has been implicated as being second-handedly important in cell proliferation and differentiation via regulation of proteins integral to these events [56]. Five years later, in 2010, Lee et al. further demonstrated the importance of modifications at or near the C-terminus of APOE by characterizing glycosylated and sialylated plasma and cell-derived APOE [55]. Several different glycoforms were identified, and a novel glycoform was discovered at Ser290 of APOE(283–299). The C-terminus of the apolipoprotein is incredibly important in lipid binding, and therefore, a glycosylation at Ser290 may have very important implications in biological mechanisms, just as the phosphorylation demonstrated by Raferty et al. may. It was also noted that while many of the glycan structures were identified, several of the more complex sites were left unidentified [55]. The difficulties and considerations associated with characterizing post-translational modifications (PTMs) in relation to AD are outlined in a 2018 review [16], and although site-specific PTM analysis continues to be a challenge, mass spectrometric analysis of glycans has made extreme improvements in the last decade [57,58,59]. With the biological implications of this work, revisiting the unidentified structures may be beneficial. Glycan analysis continues to be difficult due to glycan products typically being very large (on the order of several thousand mass units), which contributes to the idea of these C-terminus glycan products making it difficult for APOE to participate in essential lipid binding. There are several proteomic methods for analyzing glycans that would be useful in application to apolipoprotein activity analysis [60,61,62].

Immunohistochemistry-based testing and Elisa assays have both been utilized to evaluate apolipoprotein content in AD [63]. Immunohistochemistry is an invaluable tool in molecular characterization and distribution studies. However, it typically requires specific antibodies and targeted analysis. In addition, these studies supply little information regarding the absolute or relative abundance of one species to another. Mass spectrometry-based methods, on the other hand, do not require expensive antibodies and can elucidate more information regarding quantitation. MSI experiments essentially combine the two types of analysis. Distribution analysis can be accomplished without antibodies, and multi-modal imaging can be combined with other mass spectrometry or imaging techniques to develop a deeper understanding of the sample [64]. MSI and especially multi-modal imaging merging capabilities are relatively new techniques, and it would be beneficial to revisit some older mass spectrometry-based works focused on the influence of the apolipoprotein type [65,66] and APOE gene type on the disease state with MSI. An important aspect of mass spectrometry-based assays is the ability for absolute quantitation. Confirming that APOE exists in a sample is important, but being able to determine how much is integral. Few studies have focused on the development of methods with a goal of quantifying APOE content [40,67,68]. C-terminal fragmentation patterns of APOE have also been primarily studied via immunohistochemical methods [68], but a few have utilized mass spectrometry to quantify the accumulation of prominent fragments [67,68] and/or absolutely quantify APOE isoforms [40]. These utilize techniques such as multiple reaction monitoring (MRM) [40,68], which is a highly selective and sensitive lipidomic technique where a mass spectrometer is set to only monitor very specific mass/charge (*m*/*z*) ratios, allowing for the analysis of lipids present in low concentrations within complex samples [69], and MSI [67].

## 3. Lipid Peroxidation and Associated Oxidative Stress

As it pertains to AD specifically, there are thought to be two primary sources of lipid peroxidation: amyloidβ-induced lipoperoxidation of membranes and breakdown products of oxidative stress (primarily F2-isoprostanes and 4-hydroxy-2,3-nonenal (HNE)) [70]. Lipid peroxidation is a complex, non-enzymatic process that is initiated by reactive oxygen species (ROS) [71]. Increased lipid peroxidation in the AD state has been thoroughly documented [72,73,74,75,76,77,78], but mass spectrometric characterization of the two primary sources has been limited.

Amyloidβ-induced lipoperoxidation is one of the most reported potential links in lipid-relevant oxidative stress and the amyloid hypothesis of pathogenesis [76,77,79]. Mass spectrometric assays of oxidative damage to lipid membranes have shown that amyloidβ42 accelerates lipid damage [79]. Additionally, amyloidβ42 misfolds and forms fibrils (the form of amyloidβ that aggregates into plaque formations) due to oxidatively damaged lipid membranes. This amyloidβ-induced oxidation of lipid species is thought to occur primarily via the production of H_2_O_2_ by amyloidβ interaction with increased levels of Cu(II) and other metal ions in the AD brain [80,81,82]. Of these lipid by-products, HNE and isoprostanes are some of the most common [77,78,83,84]. Mass spectrometric work involving the binding of metals to synthetic amyloidβ peptides [85] has progressed the area of research toward being able to localize redox activity within tissue sections using multi-modal imaging studies, but this may require very high resolution instrumentation and advanced statistical analysis for data processing (see results and conclusions for ways forward).

HNE is an abundant aldehyde by-product of lipid peroxidation and has been linked to preclinical AD as a potential biomarker [86]. HNE is thought to stem from oxidative damage to PUFAs, particularly those that are found predominately in grey-matter-rich sections of the brain, where amyloid build-ups are also typically found [87]. A few lipidomic-based studies have characterized increased HNE in subjects with mild cognitive impairment (MCI) and advanced disease states [73,88,89,90], but there has been a lack of advanced studies in recent years focused on this breakdown product of lipoperoxidation. Isoprostanes are another common breakdown product of oxidative stress. A recent lipidomic method for early diagnoses of AD using lipid peroxidation markers in plasma samples has been reported. The work by Pena-Bautista et al. [91] focused on the development of a two-stage statistical analysis model that utilized neuropsychological evaluation and LC-based lipidomics to first differentiate between AD and non-AD groups via evaluation alone and subsequently differentiate between AD and non-AD patients based on the lipid peroxidation signature found in plasma samples. The primary predictors were isoprostanes. The combination of classic neurological evaluations and mass spectrometric data from a sample such as plasma, which is much easier to obtain than CSF or brain samples, is paramount to early diagnosis and screening. The group was able to take advantage of the fact that isoprostane markers are typically exaggerated in plasma and urine—able to partition into bodily fluids—making them key biomarkers or therapeutic targets for early intervention [92,93,94,95]. While sample preparation can be convoluted under certain circumstances, there are many published protocols for the targeted analysis of species from plasma [96,97,98]. These are typically robust, reproducible, and straightforward methods that are perfect for means of screening and diagnosis.

## 4. Inflammation

The two most prominent theories surrounding AD pathology include the amyloid plaque and the neurofibrillary tangle (NFT) hypotheses [99]. Most recently, a third pathology has arisen that essentially links the amyloid hypothesis and the NFT hypothesis: the inflammation hypothesis [99]. Acute inflammation is an important and critical response in maintaining brain homeostasis. Inflammation can act as a defense against infections and injury [99]. However, when inflammation is chronic or a disruption in the inflammatory response occurs, neurodegenerative pathology can arise. A long-term pro-inflammatory immune response (distinguished by elevated inflammation markers) has been linked to the onset and progression of many neurodegenerative disorders, including AD. Neuroinflammation appears to significantly raise the amyloidβ burden in the brain, leading to increased build-up of amyloid plaque cores, and raise the level of phosphorylation of tau, leading to increased formation of NFTs [99]. Much of the dysregulation of the immune response seen in AD brains revolves around microglia [99]. Normally, microglia observe the environment, activating only when a threat is recognized. Amyloidβ peptides are thought to be the trigger for microglial activation in the AD brain. Initially, this response aids in the clearance of amyloidβ peptides, but after some time, even with prolonged activation, clearance is no longer possible, and the peptides begin accumulating, leading to the damage of neurons [99]. While this pathogenesis is not fully understood, lipidomic-based studies have focused on the relationship between lipid metabolism and lipid expression around amyloid cores and neuroinflammation, some of which are discussed below.

Amyloid plaque formation has been one of the primary foci in AD research for decades. Initially, it was suspected that these plaques were primarily composed of toxic amyloidβ peptides; however, our previous work, as well as many others, has shown that the plaque build-ups are actually much more complex than originally thought [100,101,102]. Drugs and interventions meant to target amyloidβ aggregation have been relatively unsuccessful, and while there is no dispute that amyloid aggregation is a primary hallmark of the disease, it is obvious that there is more to plaque formations than simple peptide build-up. MSI experiments have begun to target some of the other species associated with, or localized close to, plaque formations. In 2018 and 2022, Michno et al. [103,104] utilized MSI to link amyloidβ aggregation to lipid accumulations. A positive attribute of mass spectrometry (MSI in particular) is the potential for multi-modal experimentation, mentioned briefly earlier in this review. In the 2018 Michno study, a multi-modal method for relating MSI data to classic fluorescent amyloid staining was exploited for the analysis of lipid accumulation around amyloid cores in the APP knockout transgenic mouse model. A particularly interesting finding consisted of lysophospholipids (implicated in the pro-inflammatory theory) being increased in all amyloidβ cores. Fluorescent staining has the same benefits and issues as other immunohistochemistry techniques. The fluorescent marker used for plaque cores is amyloidβ-specific, and therefore, even though it tells us little about the colocalized species, it allows for a more targeted analysis of tissue [37]. Amyloid cores, or accumulations, can be easily pinpointed, and a more targeted, selective mass spectrometric analysis can be accomplished [37]. Since then, several other studies have taken advantage of the usefulness of combining MSI data with fluorescent staining images in order to link lipid localization with amyloidβ localization [67,105,106,107]. Similar multi-modal methods have been utilized to demonstrate an increase in phospholipid accumulation and sulfatide depletion in relation to amyloidβ core localization [104,108]. Sulfatide depletion is thought to be one of the earliest events in AD onset [109,110], so advanced methods for determining the mechanisms of depletion would be paramount to early diagnoses/prevention.

An extension of multi-modal mass spectrometry comes with the pairing of MSI and higher-resolution LC/MS. Microextractions can be taken from specific areas of interest on tissue sections post-MSI and analyzed via LC/MS for more accurate mass analysis [111,112]. Emre et al. [113] combined MSI and LC/MS to analyze lipid mediators and phospholipid species in the APP knockout AD mouse model. Age-specific lipid profiles were determined based on mass spectrometry of mice aged 2–18 months. In disagreement with previous studies, pro-inflammatory and pro-resolving lipid mediators were greatly increased in older mice. However, in agreement with some other work, changes in phospholipid expression signaled some of the earliest changes in the brain parenchyma [113]. Unfortunately, few imaging-based localization studies of pro-resolving/pro-inflammatory lipid mediators have been published. Primary quantitation of these lipid species has been accomplished via LC/MS or other shotgun proteomic methods alone [114,115,116], indicating a gap in the research area. In contrast with the APP knockout mouse model analysis, in the human AD case brain, the lipid mediators responsible for resolving inflammatory events are underexpressed. Zhu et al. (2016) [117] utilized LC-MS-MS to confirm this downregulation. They determined that the expression of several pro-resolving mediators (MaR1, PD1, and RvD5) was indeed less in AD brains compared to age-matched controls. In addition, they found that the levels of PGD2 (a pro-inflammatory prostaglandin) were greatly increased in the AD case. Subsequent in vitro studies of the stimulation of the anti-inflammatory pathways by the addition of pro-resolving lipid mediators demonstrated neuroprotective activity [117]. This work supports the potential for pro-resolving lipid mediator therapy as a means of combating the lack of special mediators in the disease state. Imaging studies revolving around some of these observed mediators may be sufficient to resolve the connection between amyloid cores and colocalized lipid species.

In addition to the prolonged microglial activity leading to a chronic inflammatory response, the potential roles of gangliosides in the inflammatory hypothesis of AD have been investigated via mass spectrometry. Caughlin et al. (2015) [118] demonstrated the roles of gangliosides in Alzheimer’s disease by MSI of rat brains. Gangliosides are an important class of membrane lipids that have implications in mediating the brain’s response to injury and inflammation [118]. The altered expression of gangliosides can be linked to many disease and injury states [119,120,121,122,123]; therefore, being able to map their existence (or lack thereof) in AD model tissue is an important first step in better understanding the mechanistic roles of ganglioside expression in Alzheimer’s disease. In the Caughlin study, ganglioside expression and localization were monitored after induced amyloidβ toxicity and stroke. It was determined that GM2 and GM3 (two A-series gangliosides) were elevated in all cases (stroke, amyloidβ toxicity, and stroke/toxicity) at 3 and 21 days [118]. The innovative use of MSI, in this case, not only resolved the increase in expression but allowed the exact localization of the increase within the ischemic brain region. More recently, Kaya et al. utilized MSI technology to localize ganglioside species in plaque accumulations [124].

## 5. Conclusions and Perspectives

There are numerous published studies that have utilized mass spectrometry-based lipidomic methods for the analysis of standards, tissue, CSF, and plasma samples to help elucidate the interconnections of the various hypotheses of Alzheimer’s disease pathogenesis. This review specifically focused on studies that served to shed light on lipid-based mechanisms that are less focused on. As instrumentation continues to be able to yield more resolved information at lower concentrations of analytes and sample preparation becomes more sophisticated, additional information about lipid involvement in AD will be accessible. A paradigm shift in Alzheimer’s disease research is noticeable as of recent years. A greater focus is being put on investigating the earliest signs of AD development and potential disease biomarkers in serum and plasma. While post-mortem analysis of the brain aids in developing an understanding of how the disease progresses to end stages, it is not as helpful in determining early intervention. Plasma [125] and serum [126] lipidomes, preclinical blood markers [127], and the mitochondrial AD lipidome [128] have all been recently investigated via lipidomic mass spectrometry.

A few multi-modal imaging studies were discussed previously, but research going forward should focus more on the combination of methods to obtain a complete look at lipid involvement in AD. Extensive information exists linking lipid metabolism to amyloid build-ups and NFTs, yet clinical interventions continue to attempt to target amyloid directly when more focus should be put on the mechanisms happening in conjunction with amyloid aggregation. Tissue imaging of human post-mortem tissue samples will greatly assist with this. While animal models are integral to disease research, in the case of AD, drugs that have worked in plaque clearance in mice have not translated to human subjects. This would also make it easier to connect plasma and CSF biomarkers to brain environments. An issue facing this type of work comes with data analysis. Multi-modal imaging experiments create large amounts of convoluted data, and sophisticated machine learning and big data analysis techniques are required to analyze the data for significance. A 2020 study outlined a comprehensive analysis of omics data using artificial intelligence and advanced machine learning, proving that these issues are at the forefront of omics research [129]. These data analysis techniques have started to be used to accomplish important multivariate analysis, such as linking factors such as age, sex, and ethnicity [130] to lipid expression in AD and determining overlaps in biomarkers between different disease states [131]. Because AD disproportionately impacts some populations, linking biomarkers to factors other than just genetic predisposition could lead to important advancements in personalized medicine and intervention. Additionally, advanced multi-omics [132] techniques are being developed that allow for the integration of lipidomic, proteomic, and metabolomic studies, leading toward a more complete mechanistic understanding of the AD brain and the interconnections of multiple disease pathways.
